# A signal processing approach for enriched region detection in RNA polymerase II ChIP-seq data

**DOI:** 10.1186/1471-2105-13-S2-S2

**Published:** 2012-03-13

**Authors:** Zhi Han, Lu Tian, Thierry Pécot, Tim Huang, Raghu Machiraju, Kun Huang

**Affiliations:** 1 College of Software, Nankai University, Tianjin, China; 2 Department of Biomedical Informatics, The Ohio State University, USA; 3 Department of Health Policy and Research - Biostatistics, Stanford University School of Medicine, Stanford, USA; 4 Department of Molecular Virology, Immunology and Medical Genetics, The Ohio State University, USA; 5 Department of Computer Science and Engineering, The Ohio State University, USA; 6 The CCC Biomedical Informatics Shared Resource, The Ohio State University, USA

## Abstract

**Background:**

RNA polymerase II (PolII) is essential in gene transcription and ChIP-seq experiments have been used to study PolII binding patterns over the entire genome. However, since PolII enriched regions in the genome can be very long, existing peak finding algorithms for ChIP-seq data are not adequate for identifying such long regions.

**Methods:**

Here we propose an enriched region detection method for ChIP-seq data to identify long enriched regions by combining a signal denoising algorithm with a false discovery rate (FDR) approach. The binned ChIP-seq data for PolII are first processed using a non-local means (NL-means) algorithm for purposes of denoising. Then, a FDR approach is developed to determine the threshold for marking enriched regions in the binned histogram.

**Results:**

We first test our method using a public PolII ChIP-seq dataset and compare our results with published results obtained using the published algorithm HPeak. Our results show a high consistency with the published results (80-100%). Then, we apply our proposed method on PolII ChIP-seq data generated in our own study on the effects of hormone on the breast cancer cell line MCF7. The results demonstrate that our method can effectively identify long enriched regions in ChIP-seq datasets. Specifically, pertaining to MCF7 control samples we identified 5,911 segments with length of at least 4 Kbp (maximum 233,000 bp); and in MCF7 treated with E2 samples, we identified 6,200 such segments (maximum 325,000 bp).

**Conclusions:**

We demonstrated the effectiveness of this method in studying binding patterns of PolII in cancer cells which enables further deep analysis in transcription regulation and epigenetics. Our method complements existing peak detection algorithms for ChIP-seq experiments.

## Background

Chromatin immunoprecipitation combined with next generation sequencing technology (ChIP-seq) has been swiftly adopted as a standard technique for studying genome wide protein-DNA interaction patterns during the past four years. It is applied in gene regulation studies for identifying transcription factor targets and binding motifs, as well as in epigenetics research towards the characterization of chromatin states using various histone marks and RNA polymerase II (PolII) [1-3].

**Figure 1:**
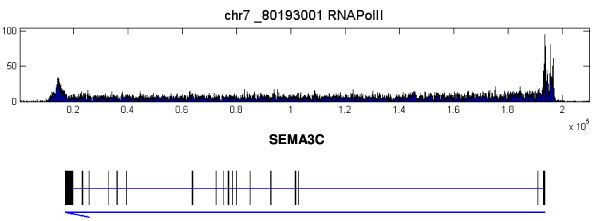
**Examples of PolII ChIP-seq data for MCF7 cell line**. ChIP-seq data for PolII binding pattern on SEMA3C in MCF7 cell control samples. The top lane shows the histogram of the PolII binding densities over a range of genome. The gene covered by this range is shown in the bottom lane. In the bottom lane, the thick bars below the gene symbol indicate exons of the gene while the blue arrow indicates its orientation. The tail and head of the arrow correspond to the transcription starting site (TSS) and transcription ending site (TES) of the gene respectively. The same arrangements are also applied to the other figures. It is apparent that PolII not only binds to the TSS regions of the gene but also form long enriched regions over the entire transcript.

PolII plays an essential role in gene transcription. During transcription, it is responsible for the synthesis of nascent messenger RNA molecules (mRNA) for protein-coding genes and microRNAs [4]. The nascent mRNAs then go through a series of processing steps including splicing to form mature mRNAs. To transcribe a gene, PolII will undergose several steps including recruitment, initiation, elongation, and dissociation [4,5]. In addition, PolII pausing and pre-mature dissociation will cause stalling of the transcription process [4,5]. Thus, accurately characterization of PolII binding patterns over the entire genome is of great importance in studying the dynamics of transcription as well as contributing to the characterization of nascent mRNA, which cannot be directly inferred from gene expression microarray or regular RNA-seq technologies since they focus on mature mRNA. However, since during transcription PolII elongates along the entire gene, the PolII binding pattern over a gene is usually not just a single peak but forms elongated regions as manifest in ChIP-seq data. PolII enriched regions can stretch to several thousands of basepairs (Figure 1). Traditionally, ChIP-seq data analysis methods rely on peak region detection algorithm to delineate genomic regions with enriched protein bindings. However, the binding pattern of PolII poses a very different paradigm of computing and in turn significant challenges. Several peak detection algorithms were developed for delineating transcription factor binding sites and the anticipated regions are short (e.g., 200-1500 bp) [6-12] thus rendering such algorithms inadequate for studying proteins with prevalent binding over the entire genome such as PolII.

While ChIP-seq data can be considered a 1-D signal over the entire genome, only a few studies explicitly take advantage of signal denoising and detection methods developed in the engineering community. For instance, in [13], wavelet denoising technique was applied to filter the ChIP-seq data to identify nucleosome distribution patterns. For histone marks, a method called SISSR was recently developed [14], which takes a multiscale approach to analyze ChIP-seq data. This approach first identifies potential regions with enriched histone patterns and then links proximal regions which are separated by short intervals as a contiguous large region. The short intervals can be considered "noise" in the genome-wide signal that can be filtered out at coarser scales.

In this paper, we also consider a ChIP-seq dataset a noisy 1-D signal stretched over the genome and apply signal processing techniques combined with statistical analysis for identifying large enriched regions in ChIP-seq data. Our approach includes three main steps. First we directly apply a signal denoising algorithm to process histogram of ChIP-seq data. Noise in ChIP-seq data originates from multiple sources including non-specific binding to the antibody, artifacts in amplification (local PCR), and sequencing reads mapping errors. These confounding noises are usually modeled as spike noise with an underlying Poisson distribution. Recently it has been shown that the non-local means (NL-means) algorithm is highly effective in signal denoising compared to other commonly used algorithms such as median filter, low-pass filtering and wavelet denoising [15,16]. It should be noted that our proposed method is based on the NL-means denoising algorithm. Since the NL-means algorithm is optimal for Gaussian noise, we apply the Anscombe transformation to the binned ChIP-seq [17]. Consequently, the underlying noise distribution model is now approximated by a Gaussian distribution. Subsequently, we apply the NL-means algorithm followed by inverse Anscombe transform. The denoised data is then compared against random model to determine the threshold for region selection based on a false discovery rate (FDR) approach. Finally, regions are selected based on the threshold, the region length, and the ratio between the peak value and the threshold.

To evaluate our method, we first test our method using a public PolII ChIP-seq dataset and compare the results with published results obtained using the published algorithm HPeak [18]. Then, we apply our proposed method on PolII ChIP-seq data generated in our own study on the effects of hormone on breast cancer cell line MCF7. We compare the long segments obtained when tested against both MCF7 cell line and the MCF7 cells treated with 17-estradial (E2) hormone. The results demonstrate that our method can effectively identify both long enriched regions in ChIP-seq datasets and complement existing peak finding algorithms for a variety of potential wide applications such as histone mark binding pattern study.

## Methods

### Data selection and pre-processing

To test our method, we first downloaded a ChIP-seq dataset for PolII on a prostate cancer cell line (LnCap) after treatment with an androgen receptor agonist R1881 (GSM353618) from NCBI Gene Expression Omnibus (GEO). The dataset was generated using Illumina Genome Analyzer II (GAII) sequencer and was analyzed using the HPeak algorithm [3]. In addition, we apply our algorithm in PolII ChIP-seq data generated in our research on breast cancer using MCF7 cell line. The accession numbers are GSM529981 (MCF7 control), and GSM529982 (for MCF7 treated with 17-estradial (E2)). For all the datasets, we downloaded the sequence mapping results (using the Eland algorithm provided by Illumina Inc.) and then generated histogram with selected bin size.

### Introduction of NL-means algorithm and parameter selection

The NL-means algorithm was originally developed for image denoising [15] and was also applied to 1-D signals (including acoustic signals) [19] and video [20]. The details of the original NL-means algorithm are given in [15]. Here we briefly describe the essential formulation and the salient parameters. Basically, for each data point, its value is replaced by a weighted average of data points over the entire signal such that points with similar neighbourhoods are given higher weights. It has been shown that the NL-means algorithm is optimal for Gaussian noise. In addition, using weighted averaging for points with similar neighbourhoods is suitable for ChIP-seq experiments since the pattern of protein binding are considered similar across many regions of the genome.

**Figure 2:**
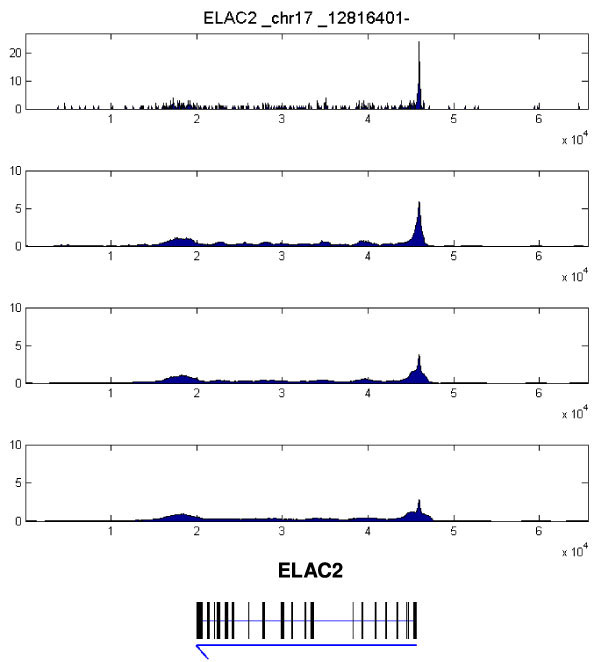
**Denoising on PolII ChIP-seq data for MCF7 cell line using NL-means algorithm**. This region is over the gene ELAC2. Different parameters for the NL-means algorithm are tested on the ChIP-seq data. The second panel uses the parameter sets used in the rest of this paper (***R***=**10**(***bins***),***L***=**15**, ***and σ***=**10**)

Formally, given a signal with data points *X*={*x_i_*,*i *= 1,...,*N*}, the filtered value at *x_i _*is defined as $NL\left[x_{i}\right]=\overset{N}{\underset{j=1}{\sum }}w\left(i,j\right)x_{j}$, where is a difference measure between *x_i _*and a neighbouring point *x_j _*under the constraints w(*i*,*j*)≥0 and $\overset{N}{\underset{j=1}{\sum }}w\left(i,j\right)=1$ Specifically, $w\left(i,j\right)=\frac{exp\left(-||N\left(x_{i}\right)-N{\left(x_{j}\right)||}^{2}/\left(2\sigma^{2}\right)\right)}{Z\left(i\right)}$, where is the normalizing factor, $Z\left(i\right)=\underset{j}{\sum }exp\left(-\frac{||N\left(x_{i}\right)-N{\left(x_{j}\right)||}^{2}}{2\sigma^{2}}\right)$, and $N\left(x_{i}\right)$ denote a fixed-size neighbourhood centred at the position *i*. In practice, searching for similar neighbourhood patterns over the entire genome is not feasible. Instead, a parameter specifying the range of search is needed. In summary, the NL-means algorithm requires three parameters, the size of neighbourhood the range of search and the weight parameter We evaluated a series of parameter combinations (Figure 2) and in this study we use ***R ***= 10(*bins*) ***L ***= 15, *and ****σ ***= 10

### Anscombe transformation

Since NL-means algorithm is optimal for Gaussian noise while the noise in ChIP-seq data is usually modelled using Poisson distribution, we apply Anscombe transform to the data first. Anscombe transform is a commonly used variance-stabilizing transformation that transforms a Poisson distribution random variable into one with that is close to a Gaussian distribution [17,21]. The Anscombe transformation on data point is defined as follows: $y=2\sqrt{x+3/8}$ and the inverse Anscombe transform is given by *x*=(y/2)^2^-3/8.

### FDR based approach

To select a threshold for region selection, we designed a FDR approach based on *B *random simulations. Given the histogram of the ChIP-seq data with *M *bins and the reads over each bin as *r_i _*(*i = 1, 2,..., M*), we take the following steps:

1. In the b-th round of simulation (*b = 1, 2,..., B*), we randomly (Poisson distribution) position the same amount of sequencing reads for each chromosome as in the original data and then apply the NL-means algorithm. The reads over the i-th bin is denoted as $r_{ib}^{\text{*}}$.

2. Then for the i-th bin in the histogram, the ratio that the observed data is less than simulated data is recorded as ${\text{p}}_{\text{i}}=\overset{\text{B}}{\underset{\text{b}=1}{\sum }}\text{I}\left({\text{r}}_{\text{i}}<{\text{r}}_{\text{ib}}^{\text{*}}\right)$. I(A) takes value 1 (resp. 0) if A is true (resp. false).

3. For the b-th round of simulation, we treat it as a "true" signal and compare it with rest of simulated data. For the *i*-th bin, we compute $p_{ib}^{*}=\overset{B}{\underset{b^{\prime }=1}{\sum }}I\left(r_{ib}^{*}<r_{ib^{\prime }}^{*}\right)$Then for a threshold *p_cut_*, the number of false peaks in this round of simulation is $d_{b}=\overset{M}{\underset{i=1}{\sum }}I\left(p_{ib}^{*}<p_{cut}\right)$

4. The false discovery rate for the cutoff *p_cut _*is $FDR\left(p_{cut}\right)=\frac{B^{-1}\overset{B}{\underset{b=1}{\sum }}d_{b}}{\overset{M}{\underset{i=1}{\sum }}I\left(p_{i}\leq p_{cut}\right)}$.

Since *p_cut _*is a function of the region height, we obtain the FDR for selected threshold on height. Then for each detected region based on the FDR approach, we also calculate the ratio between the peak value and the threshold.

## Results

### Comparing with HPeak for detecting PolII enriched regions in prostate cancer

**Figure 3:**
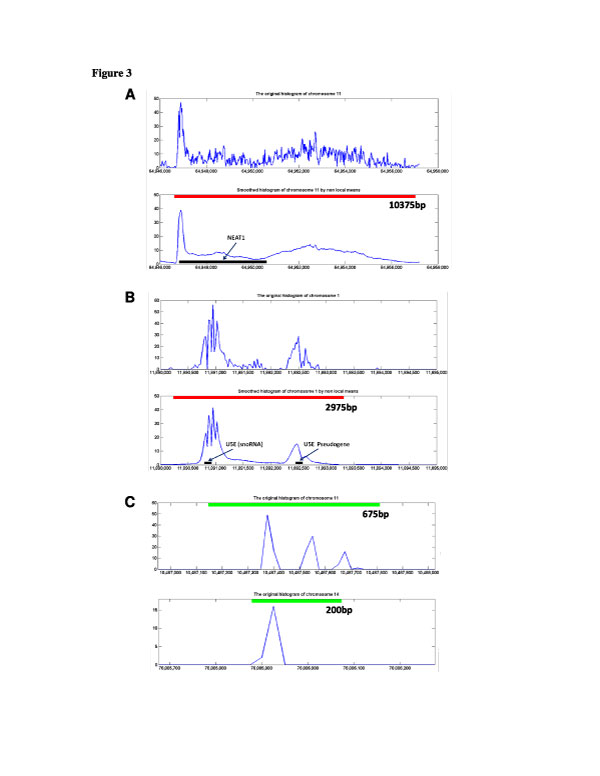
**Comparison of HPeak and the proposed algorithm in detecting regions on the prostate PolII ChIP-seq data (parameters of 25 bp for bin size, 0.01 for FDR and 6.5 for Peak-to-threshold ratio)**. The bin size is the same between the two algorithms. A: A region detected using the proposed algorithm but not by HPeak. Top: the original histogram of the ChIP-seq data. Bottom: the NL-means filtered data and the detected regions (marked by the red bar). The length of the detected region (10375 bp) is marked under the red bar. A gene (NEAT1, marked by black bar) is covered by this region. B: Another region detected using the proposed algorithm but not by HPeak. Top: the original histogram of the ChIP-seq data. Bottom: the NL-means filtered data and the detected regions (marked by the red bar, 2975 bp long). A snoRNA gene (U5E) and its pseudogene (marked by black bars) are covered by this region. C: Two regions detected by HPeak but not the proposed algorithm. The HPeak detected regions are marked using green bars (the lengths are 675 bp and 200 bp, respectively). We examined all regions that were missed by the proposed algorithm under this set of parameters, all regions contain short spike-like peaks as shown here.

We first apply our method to a published PolII ChIP-seq data in prostate cancer model (GSM353618). We compare our results with the published 15,833 PolII enriched region detected using HPeak [3,18]. As shown in Table 1, when FDR = 0.01, our method show a high overlap with published results with overlap ratio (comparing to HPeak) of 80-100%. We then inspected many of regions that were detected using our method but were missed by HPeak. We found many of them correspond to potential transcription starting sites (TSS) for unknown genes or non-coding RNA genes. Figure 3A-B show examples of regions detected using our method using 0.01 for FDR and 6.5 for Peak-to-threshold ratio but were missed by the HPeak algorithm even though they imply the potential transcription activity for the covered genes. One of them is a snoRNA gene (U5E). Figure 3C shows examples of regions that were detected by HPeak but were missed by our method. In fact, using this setting, all regions missed by our method contain narrow peaks (covers one to two 25 bp bins). This observation suggests that the regions detected using our method can provide addition information regarding gene transcription and annotation. Further detailed annotation on these new regions is currently being carried out by integration with other information including CpG island distribution, DNase I hypersensitivity, and H3K4me2 binding (ChIP-seq).

**Table 1:** Comparing our method with HPeak (bin size 25 bp).

FDR	Peak/Threshold Ratio	# of Regions	Ratio of Overlap with HPeak (%)
0.05	5	108393	100

0.05	10	35243	100

0.03	9	24213	100

0.03	10	20421	93.09

0.02	8	21699	98.11

0.02	10	15612	80.90

**0.01**	**6**	**22859**	**100**

**0.01**	**6.5**	**20358**	**96.65**

**0.01**	**8**	**15302**	**80.01**

### Detecting large regions with PolII enrichment in breast cancer cell line MCF7

**Figure 4:**
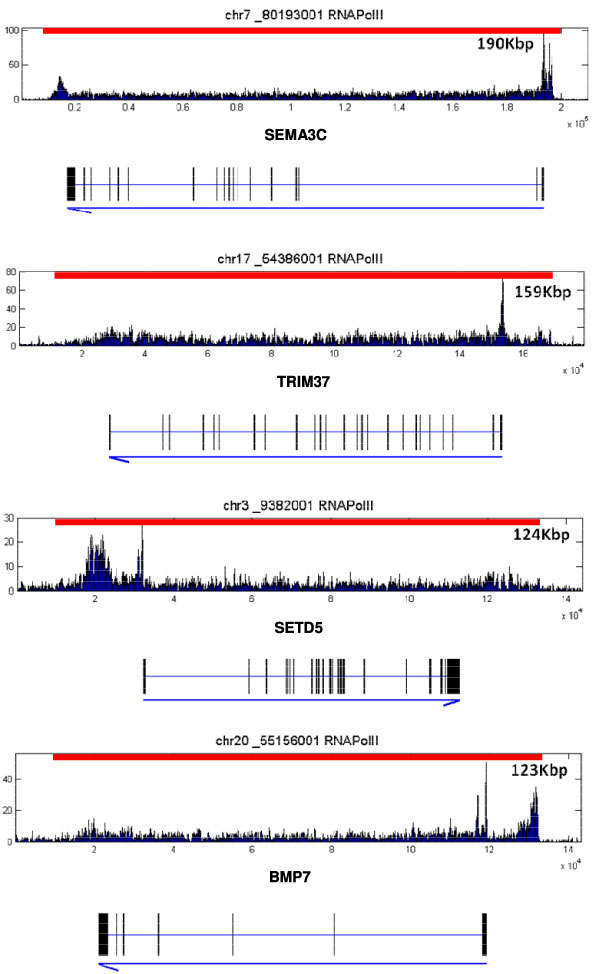
**Examples of long regions of PolII binding detected using our algorithm**. Red bars indicate the detected regions. The segments are shown above the patterns over corresponding genes. The lengths of the segments are listed in the figure. The four genes (from top to bottom) are SEMA3C, TRIM37, SETD5, and BMP7.

We then applied our method in the PolII ChIP-seq datasets for the breast cancer cell line MCF7 using large bin size (1,000 bp). As shown in Table 2, pertaining to MCF7 control samples we identified 5,911 segments with length of at least 4 K bp (ranging from 4,000 bp to 233,000 bp); and in MCF7 treated with E2 samples, we identified 6,200 such segments (ranging from 4,000 bp to 325,000 bp). Some examples of the long regions detected in MCF7 control samples are shown in Figure 4.

**Table 2:** Long enriched regions identified in PolII ChIP-seq data in MCF7 cells using bin size of 1,000 bp.

Sample	# of regions with length ≥ 4,000 bp	# of regions with length ≥ 10,000 bp	Maximum length (bp)
MCF7 control	5,911	1,992	233,000

MCF7 + E2	6,200	2,310	325,000

## Discussion

**Figure 5:**
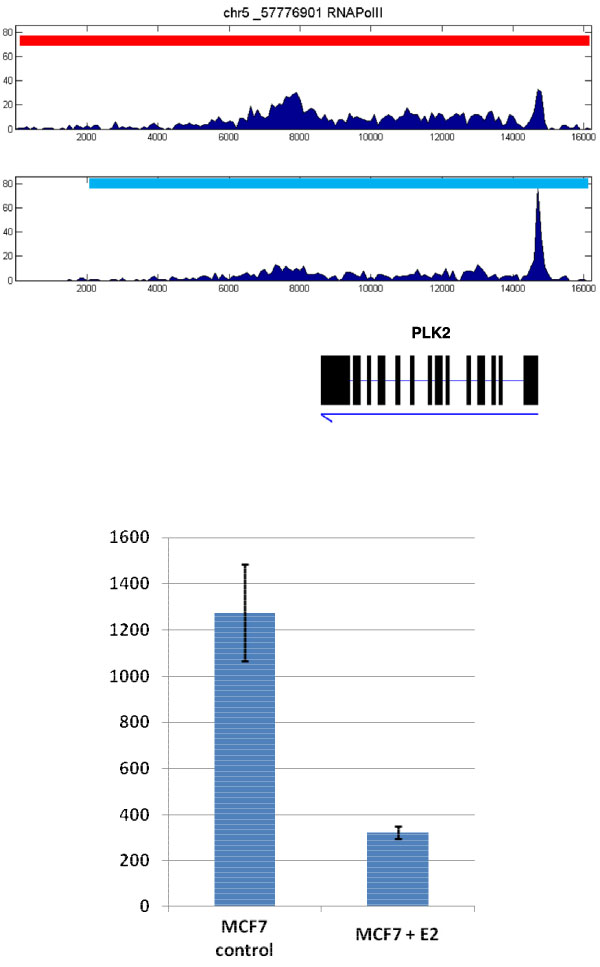
**The PolII binding patterns and expression levels for PLK2 gene**. Top: the PolII binding patterns for PLK2 gene in control (first lane) and E2 treated samples (second lane). PolII shows a higher peak for the E2 treated sample but lower total amount of binding over the transcript. Red bar indicates the 16 Kbp region detected using our method in MCF7 and blue bar indicates the 14 Kbp region detected in MCF7+E2. Bottom: the expression levels of PLK2 gene in the two different conditions (n = 4). Error bar is for standard deviation.

Identification of long segments for PolII binding are important for further investigation for understanding gene transcription regulation as well as potentially discovery novel transcripts and alternative promoters. For gene transcription, while PolII binding density at promoter around the TSSs was considered to determine gene transcription levels, recent studies show that the density of PolII binding on gene body is also critical [5,22]. We also observed such phenomena using the above identified segments. For instance, as shown in Figure 5, a segment of 16,000 bp has been identified over the transcript of the gene PLK2 on human chromosome 5. The MCF7 control sample has more sequencing reads over this region than the MCF7 sample treated with E2 sample (958 vs 454 reads with similar amount of total reads in chromosome 5 between the two samples). Although, the height of the "peak" at the TSS region in the MCF7 control sample is lower than that in the MCF7 E2 treated sample, the total transcription level (measured by Affymetrix gene expression array) is still higher in MCF7 control by a factor of 3.95-fold (Student t-test *p *= 3.872 × 10^-6^).

The above observations suggested that ChIP-seq technology can reveal potential new insights and principles in biology. The method presented in this paper will contribute to such discovery efforts. Nevertheless, this method can be improved in several aspects. First, currently the parameters for NL-means algorithm are fixed. In practice, the user may focus on enriched regions of certain length and this could lead to the change of these parameters. Therefore, a multiscale approach is preferred. Second, an important utility of ChIP-seq data analysis is to compare enriched regions between different samples such as ChIP sample versus its input control or control sample versus drug treated sample. Currently we are implementing a Fisher's exact test based approach to enable such comparison. Last but not the least, this method can be applied not only to PolII ChIP-seq data but can also be used for analyzing other data such as histone mark ChIP-seq data for integrative genomic analysis. Currently we are also expanding our study to integrate ChIP-seq data for important histone marks including H3K4me2 and H3K27me3 in an epigenetic study.

## Conclusions

In this paper, we propose an enriched region detection method for ChIP-seq data to identify long enriched regions by combining a signal denoising algorithm with a FDR approach. Our method complements existing peak detection algorithms for ChIP-seq experiments. We demonstrated the effectiveness of this method by studying the binding patterns of PolII in cancer cell lines which enables further deep analysis with applications in transcription regulation and epigenetics.

## Competing interests

The authors declare that they have no competing interests.

## Authors' contributions

ZH implemented the algorithm and carried out all tests. LT designed the FDR test. TP advised on the implementation of NL-means algorithm. TH provided the breast cancer PolII data. RM conceived the idea of using NL-means for ChIP-seq denoising. KH led the project including development of the idea of signal processing approach, design of all experiments, interpretation of the results and writing of the manuscript. All authors edited the manuscript.
